# Spruce vs. pine did not impact soil organic carbon density but strongly affected a functionally important dwarf shrub in a boreal production forest system

**DOI:** 10.1371/journal.pone.0320877

**Published:** 2025-04-15

**Authors:** Stein Joar Hegland, Emma E. Olsson, Martin Stensaker, Jakob Gilhuus, Ben Snook, Knut Rydgren, Mark A. K. Gillespie

**Affiliations:** Department of Civil Engineering and Environmental Sciences, Western Norway University of Applied Sciences, Sogndal, Norway; Institute for Biological Research, University of Belgrade, SERBIA

## Abstract

In the boreal zone, forestry is one of the most influential drivers of land-use changes. Tree species selection is a management decision which may strongly affect environmental factors that subsequently impact many ecosystem properties including soil carbon storage and functional important species. We used a production forest system in Kaupanger, Western Norway, where Scots pine has been the main timber species and Norway spruce was introduced by forestry. We selected 10 pairs of production stands of spruce or pine with identical productivity index and age for controlled comparison of tree species identity effects on a) soil organic carbon (SOC) density and b) performance of the functionally important bilberry (*Vaccinium myrtillus* L.). We sampled soil from three depths (5, 15 and 25 cm) at three sampling points per stand for the SOC density analysis, and recorded cover, size, and reproduction of bilberry in five plots per stand. SOC density did not differ significantly between stands of spruce or pine, but pine stands had the highest density of SOC at 5 cm whereas the spruce stands had the highest SOC density at 15 cm depth. The performance of bilberry differed strongly between stand types: the predicted cover of bilberry in pine stands was 47% compared to 5% in spruce stands; the plant size (i.e., dry mass of aboveground ramets) was more than double as large in pine stands; and in spruce stands only one of 50 examined plots had any berries, compared to an almost ubiquitous berry supply in pine stands. Tree species identity appeared to have a neutral effect on the density of SOC in forest soils but had a strong negative effect on all recorded performance variables of the functional important bilberry plant. Spruce stands should be managed for improved light condition to improve habitat quality for dwarf shrubs and its associated biodiversity.

## Introduction

Global land use changes are the largest threat to biodiversity and the second biggest driver of carbon emissions [1,2]. Forests are a particularly important ecosystem in this context, covering approximately a third of the global land area, containing ca. 40% of the total organic global carbon [3], and supporting perhaps as much as 80% of world’s species diversity [4]. The management of forest ecosystems is thus crucial to solving issues related to both the climate and biodiversity crises. Globally, more carbon is stored in biomass and soils than is contained in the atmosphere [5] and forests may represent an important carbon sink that sequester a significant proportion of greenhouse gas emissions [6]. Forest soils may store as much as half of all terrestrial carbon stocks [3], and in the boreal forest, which represents about one third of global forests 60–80% of carbon stocks are contained in soils and dead matter [3,6–8]. Consequently, management of boreal forest for carbon storage should have an extra focus on soil organic carbon (SOC) and dead organic matter.

About two-thirds of boreal forest is managed by forestry [9]. Generally, the conversion of boreal old-growth forest to production forest in Fennoscandia is associated with altered tree species composition from mixed-species forests to forests often dominated by Norway spruce (*Picea abies L.,* hereafter spruce) by means of clear-cutting and planting. These forests are often cultivated in denser monocultural and even-aged younger stands, which generally have low light availability and thus a reduced structural diversity and cover of the understory vegetation layers [10–13]. In Norway, the government also subsidizes denser planting as a climate mitigation measure [14] and Norwegian law is structured to impose planting of mainly spruce [15,16]. In western Norway, it is mainly birch- and pine-dominated forest that has been converted to spruce plantations [16]. The local to large-scale ecosystem alterations from this type of forestry may ultimately affect many ecological processes, including carbon storage and biodiversity, since many forest-dwelling organisms are affected by light conditions and associated forest characteristics [11].

Coniferous dominated forest often contains higher SOC-stocks than deciduous forests because of slower decomposition rates [8,16,17]. Some studies from Fennoscandia have also found that soils of spruce stands may contain more soil organic matter (SOM), which are directly correlated with SOC, than soils in Scots pine (*Pinus sylvestris L.*; hereafter pine) forest [18–20]. This have led to the suggestion that spruce may be a better tree selection choice for optimizing carbon storage through forestry [21], although studies that control for important environmental- and stand-related variables of the impact of tree species identity on SOC are rare. Spruce planting also make forests denser, and theory may predict that more stems and a larger basal area would increase carbon stocks in spruce forests, although some empirical evidence point to no effect of denser stands on carbon stocks [3]. Moreover, species diversity may decrease when spruce stands replace pine forests, particularly for vascular plants and their associated consumers, which are directly or indirectly a result of less light reaching the understory in spruce forests [11]. The functionally important dwarf shrub bilberry (*Vaccinium myrtillus* L.) is frequently used as a model species for ecological impact studies in the boreal forest because it is a ubiquitous key species for a wide range of interacting species [22–26], and because forestry-induced changes in forest structure may reduce the cover of bilberry up to 50% [12,27]. However, forestry inventory data in Norway have pointed to a potential increase in the cover of bilberry over the last decade [28], and coupled with the potentially higher density of SOC in spruce dominated stands [e.g., 21], may suggest that the large-scale conversion from pine to spruce stands is not so influential in Norwegian production forests as elsewhere in Fennoscandia. However, as studies from neighbouring countries [e.g., 12] showed that such pine-to-spruce conversion may have huge impacts on important biodiversity-components like dwarf shrubs and associated species, additional studies are required to resolve the contradictions.

We utilized a system managed for forestry in Kaupanger in the inner Sognefjord region, Western Norway to investigate how production stands dominated by the native Scots pine or the introduced Norway spruce impacted indicators of biodiversity and ecosystem processes. The forest in the study area may reflect ecological conditions in relative climatic mild boreal to hemiboreal forests in Northern Europe. There is still relatively little published on how the large-scale conversion from spruce to pine-dominated forest affects many ecosystem properties and therefore we aimed at investigating the effects of tree species identity on SOC density and bilberry performance by using areas where spruce had been introduced by planting next to managed pine stands as a natural semi-controlled experiment. We, selected pairs of stands with identical ecological conditions and age to reduce environmental variability and increase the predictive power in investigations of forestry impacts (i.e., tree species conversion), similar to long-term field experiments [e.g., 20,29].

To compare spruce vs. pine stand effects on ecosystem processes and biodiversity, we used two main indicators: a) SOC density to enable comparison of the relative soil carbon storage potential, and b) the performance (cover, size, and reproduction) of the functional important bilberry to estimate impact on forest floor vegetation and interacting species. Based on previous studies [10,12,18,21], we expected that SOC density would be highest in spruce-dominated stands whereas bilberry would perform best, in terms of cover, size and reproduction, in pine-dominated forest.

## Materials and methods

### Study area

**Fig 1:**
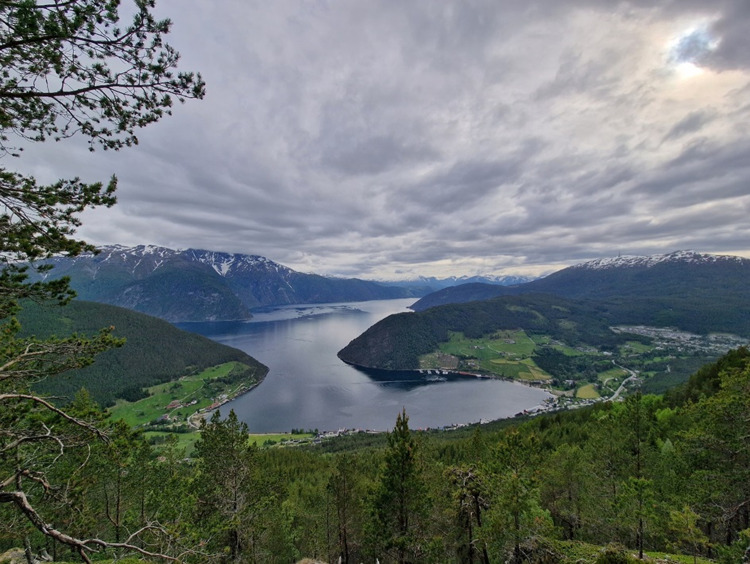
Overview of Kaupanger with the settlement by the Sognefjord, surrounded by forest areas dominated by coniferous forest from sea level to ca. 900 masl (indicated by snow at the picture). The study sites are largely situated in the centre right of the picture. Photo: Stein Joar Hegland (first author).

The study was conducted in a forest area at the Kaupanger peninsula (Fig 1) in the inner Sognefjord, Norway (61.2°N, 007.2°E), which is largely characterised as a cold temperate climate of the boreal zone [30]. The soils are uniform, acidic, and podzolic, across the study area, caused by the combination of a anorthosite bedrock and moraine sediments [31]. In a multi-site study at 100, 500 and 900 masl in the same are we found that pH varied from 3.7 to 4.6 with a mean of 4.2 (Unpublished data, Knut Rydgren, Mark A. K. Gillespie and Stein J. Hegland). The study area comprises boreal forest dominated by pine, spruce, and birch (*Betula pendula* Roth and *B. pubescens* Ehrh.) in the tree layer, and bilberry, lingonberry (*Vaccinium vitis-idaea* L.), and crowberry (*Empetrum nigrum* L.) and other dwarf shrubs in the field layer. According to the forest inventory maps from the largest landowner at on the peninsula, Kaupanger Hovedgård, the production forest contains spruce stands that are up to 100 years old, and pine stands up to 160 years old [32]. While most spruce trees are not older than 60 years, there is a considerable number of older pine trees in the higher elevation forest (>500 masl) of the study area (pers. obs. S.J. Hegland). Dendrochronological studies at ca. 700 masl in forests surrounding the study sites have identified pine trees up to at least 450 years old [33] and a considerable amount of this mid-elevational forest has been protected in this area [34]. The study was performed in close collaboration with the landowner, Kaupanger Hovedgård, that gave authorisation to use the roads and the stands for the current research.

### Sampling design and data collection

In 2021, we established 10 study sites in production forest between 185 and 615 masl, based on the assumption that these sites could reflect a natural experiment. The paired stands of pine and spruce in each site was selected to have identical productivity index, stand age and logging class based on forest inventory maps [32], and we also aimed at having stands of equal size, tree density, and elevation but tested for the latter variables (see data analysis). The selected pairs of stands varied across but not within pairs with respect to table values from the forestry inventory: 30–100 years stand age, II to V logging class (i.e., all classes in the Norwegian forestry classification system except clearcuts), 11–20 in site productivity index (i.e., low to intermediate-high productivity, but not the lowest and highest productivities), 0.6 to 9.9 hectares (ha) stand area, 2.9 to 25 stems/ha. The production forest in the area, both pine and spruce, has largely been regenerated by planting trees with approximately 2 m distance (pers. comm. Torbjørn Hegland, retired forest manager). We thus avoided the potential confounding effects of environmental conditions between stands by the paired design for the impact of stand type (i.e., the tree-species identity effect), but let other variables vary across pairs. Confounding factors such as productivity (site or productivity index in forestry) or forest age and other environmental variables may often be an issue in larger, but uncontrolled, study designs using regional or national forest inventory data [29].

In each stand in 2021, we designated a 20 × 20 m block, at least 5 m away from any edge, and selected three random sampling points for soil sampling. Sampling points were rejected and replaced by the next available position if there was a tree, stone or large dead tree at the point, or a previous sampling point < 3 m away. At each sampling point we made a soil pit of ca. 30 cm deep and sampled soil at by inserting 120 cm^3^ tube (h= 6 cm, radius= 2,5 cm) horizontally into the pit wall with the centre of the cylinder. Samples were taken at the forest floor layer and two mainly mineral soil layers, at 5, 15 and 25 cm depths below the moss (i.e., bottom) layer following [18] and [16]. The soil samples (N = 180) with this set volume were each deposited into a paper bag and transported to the laboratory for further analysis. At each sampling point we recorded the slope gradient to add to the environmental variables that were available in maps and forest inventory tables.

In 2022, we placed five randomly selected plots of 0.5 × 0.5 m in a new designated 20 × 20 m block within each stand, at least 5 m away from any edge, for sampling of bilberry performance in the stands (N = 100). Plot positions were randomly selected and rejected following the same criteria as for soil sampling. In each plot, we visually estimated the percentage cover and counted the number of berries. Lastly, we selected four aboveground shoots of bilberry, towards each corner of the plots and we measured their height from ground to canopy, the number of annual shoots and the stem diameter at base that together were used to estimate plant ramet size (see Variable calculations). For each plot we recorded light conditions in stands by using a lux meter (Voltcraft LX-10) at ca. 0.5 m height facing south by each of five study plots.

### Variable calculations

The soil samples were dried at 105°C for 24 hours to constant weight and then weighed. Bulk density was determined by dividing the dry mass of the soil sample by its total volume. Then soil samples were sifted using 2 mm mesh width to obtain the fine fraction and the mass of coarser material (> 2mm) was weighed separately to obtain the coarse fraction. Soil organic matter (SOM %) was measured by loss-on-ignition by burning the samples of the fine soil in crucibles at 550 °C for six hours following Krogstad [35]. SOM was the multiplied by the conversion factor, 0.5 following the recommendations of Pribyl [36] to estimate the organic C_*content fine*_ (%) and thereafter SOC density as [37]:


$$SOC_{density}=SOC_{content,fine}\times \left(1-massproportion_{coarse}\right)\times p$$

where SOC_density_ is the mass of organic carbon in a given volume (g/cm^3^ mass proportion_coarse_ is material > 2mm divided on the whole sample (%), and *p* is the bulk density of the soil sample (g/cm^3^). The loss-on-ignition approach is easily applicable and gives comparable results to a TOC-analyser for a fraction of the price and time [36,38].

Plant size of the four sampled bilberry ramets per plot were estimated using the formula of Hegland et al. (2010) which non-destructively calculates the dry mass of bilberry ramets with a precision of ca. 94% [39]:


$${\text{log}}_{\text{2}}\left(\text{DM}\right)\text{= 1}{\text{.41700 x log}}_{\text{2}}\left(\text{DS}\right)\text{+ 0}{\text{.97104 x log}}_{\text{2}}\left(\text{H}\right)\text{+ 0}{\text{.44153 x log}}_{\text{2}}\left(\text{AS + 1}\right)\text{- 7}.52070$$

where DM is the dry mass, DS is the stem diameter, H is the ramet height, and AS is the number of annual shoots. The approach is beneficial because it can combine several biometric measurements into a single plant-size variable with minimal impact on investigated populations and without introducing biases in multiannual research [40].

### Data analysis

First, we used paired t-test to assess whether the physical environment differed between the paired stand types of spruce or pine was similar as we had planned for in the study design. To test for differences in light conditions measured by the lux meter we used the Wilcoxon rank sum test due to non-normality. Inspection of the paired differences revealed two extremely large outliers due to unusually high light readings in two spruce stands, which may sometimes be a problem when using a lux-meter for light conditions (pers. obs. S.J. Hegland), and we therefore performed this test without the two affected pairs.

Second, we performed modelling to test our main hypotheses on effects of stand type on SOC and bilberry performance. We used Linear mixed modelling (LMM) where the response variables were normally distributed (or could be transformed to approximate a normal distributed), and Generalised linear mixed modelling (GLMM) where they were non-normal. The mixed modelling approach was taken because it was necessary to account for the hierarchical sampling design. We used the *glmmTMB* package [41] with random intercepts for sampling points or plots within stands and we assessed modelling assumptions (normality of residuals and random effects, homogeneity of variance) using the simulation approach employed by the *DhARMA* package [42]. All modelling assumptions were satisfied unless stated below.

For models of SOC density, SOM (%) and bulk density, we tested the effect of stand type and soil depth by including these factors and their interaction as fixed effects, and sampling point nested within site as random effects. If the interaction between stand type and soil depth was not significant, we removed it from the model to interpret the main effects and confirmed that the subsequent model was superior by means of likelihood ratio tests. Both bulk density and SOC were normally distributed and were therefore modelled with a Gaussian error distribution (LMM), whereas SOM as a percentage was modelled with GLMM with beta distribution and logit link. This is an appropriate distribution to use for proportion data [43] and resulted in normally distributed residuals. However, all models suffered from residual patterns with heterogenous variance and overdispersion, and these patterns were addressed by allowing the dispersion parameter to be dependent on stand type, soil depth and location [44].

We assessed differences in bilberry ramet size as dry mass between stand type with an LMM with stand type as the fixed effect and plot nested within site as random effects. Dry mass was log_2_ transformed to normalise the residuals [see 39 for more details on the modelling of dry mass] and the model was fitted with a Gaussian error distribution. Cover values were recorded as percentages at the plot level, so the random structure was reduced to site, and the beta distribution was used to parameterise the model as above. Due to a high number of zeros, particularly in the spruce stands, the model was constructed as a zero-inflated GLMM with stand type as a fixed effect in the zero-inflated part to model the probability of zero cover. This effectively splits the model result into two parts: the zero-inflated part of the model, analogous to a binomial regression model of the probability of bilberry plants occurring in a plot, and a “conditional” part that models the differences in non-zero observations in the data. Berry numbers were recorded as counts at the plot level and contained many zeros, particularly in the spruce stands where only one plot produced berries. Therefore, it was not possible to adequately model the reproduction variable, and we thus compared the stand types with descriptive statistics. All statistical testing were conducted in the R programming environment [v4.3, 45].

## Results

### Descriptives of stand types

The paired pine and spruce stands had very similar physical environments except that pine stands had more than four times as much light reaching down to the understory as spruce stands (Table 1). The similarities in most of these physical characteristics was as expected as we designed our study to take into account productivity index, logging class and associated variables.

**Table 1:** Physical characteristics of the ten paired stands of pine and spruce in Kaupanger, Western Norway based on values from forestry inventory maps or field recordings, including the results of paired t-tests (for normally distributed variables) and paired Wilcoxon signed rank tests (for non-normally distributed variables.

Stand values	Pine mean	Spruce mean	Test statistic
**Stand age** (map: yr., N=20)	55.0 (± 1.6)	58.5 (± 2.0)	t = -1.48
**Stand area** (map: ha, N=20)	31.8 (± 2.1)	26.3 (± 1.5)	t = 0.82
**Elevation** (gps: masl; N=20)	368 (± 12.8)	358 (± 13.1)	t = 0.11
**Slope** (compass: °s; N=60)	9.3 (± 1.1)	12.7 (± 1.4)	t = -1.19
**Tree density** (map: da; N=20)	107 (± 6.0)	123.5 (± 5.3)	t = -1.06
Ligh availability* (lux; N=80)	4400 (2267)	745 (589)	V = 36**

* data from two paired stands were removed as outliers (see data analysis); **p < 0.01; In parentheses: standard errors or interquartile range (lux).

### Soil organic carbon in spruce vs. pine stands

**Fig 2:**
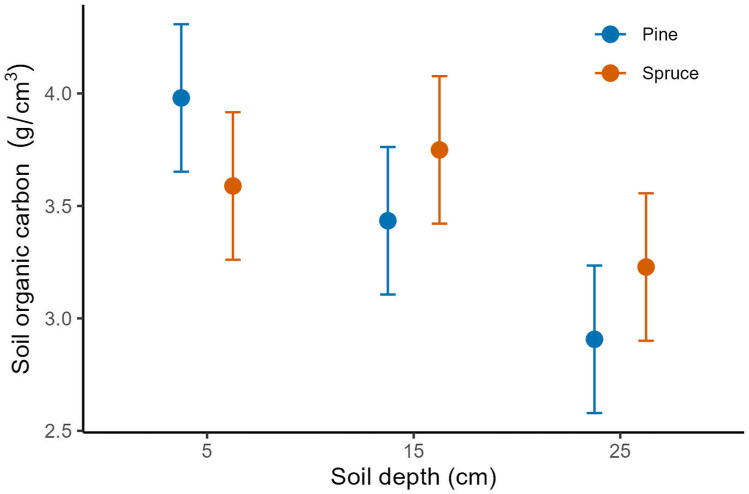
Mean soil organic carbon density (SOC) at the three soil depths within pine and spruce stands, predicted from a Linear mixed effects model with a significant interaction between soil depth and stand type (p < 0.001). Errors bars are 95% confidence intervals.

In pine stands SOC density decreased linearly with soil depth whereas in spruce stands it was highest at intermediate depth (i.e., 15 cm; Fig 2) as shown by the significant interaction (Table 2). The difference in SOC density between stand types (main effect) were minute (Fig 2; pine mean 3.49 ± 0.87 SD, spruce mean: 3.52 ±. 0.75 SD), but there was an overall decline in SOC density from 5 to 25 cm depth (5 cm: 3.80 ± 0.89 SD, 15 cm; 3.61 ± 0.73 SD, 25 cm: 3.1 ± 0.65 SD), irrespectively of stand type.

**Table 2:** Analysis of deviance tables for the generalised linear mixed models of the three measures included in our analysis on soil organic carbon.

	Soil organic carbon			Bulk density			Loss-on-ignition		
	χ^2^	df	p-value	χ^2^	df	p-value	χ^2^	df	p-value
Stand type	2.78	1	0.096	4.06	1	0.044	6.46	1	0.011
Soil depth	49.01	2	<0.001	181.9	2	<0.001	102.6	2	<0.001
Stand type x Soil depth	14.29	2	<0.001						

Type III Wald χ^2^ tests were used where the interaction was significant. Type II tests were used when the interaction was removed.

When analysing the components of SOC density separately (Table 2), the impact of stand type did not depend on soil depth (no interaction) for bulk density (g/cm^3^) or loss-on-ignition (%), but for both variables there were significant differences between both stand type (p < 0.05) and soil depth (p < 0.001). Bulk density increased with soil depth and was significantly lower in spruce stands, whereas loss-on-ignition in the soil showed the opposite pattern (Table 2, Fig S1 and S2 in S2 Appendix; S1 Appendix).

### Bilberry performance in spruce vs. pine stands

**Fig 3:**
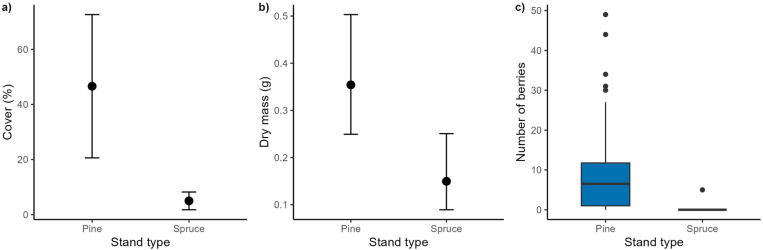
Differences for the three bilberry performance measures between stand types: a) plant cover predictions conditioned on both the fixed effect (stand type) and zero-inflation component of a zero-inflated generalised mixed effects model, b) plant size (mean aboveground dry mass of ramets; back transformed) predicted from a linear mixed effects model, c) boxplot of berry numbers per plot (not modelled due to very heterogenous data). Errors bars in a) and b) are 95% confidence intervals.

Dry mass and cover of bilberry both differed significantly between the stand types (Table 3). Pine stands had substantially higher bilberry cover with a predicted mean of 47% vs 5%; in spruce stands (χ^2^ = 53.3, p < 0.001; Fig 3a). Bilberry was present in 47 of 50 plots in pine compared to only 21 plots in spruce stands, and accordingly the model for cover (zero-inflated part) showed that spruce stands had much larger probability of having plots with no bilberry presence (probability of 0.06 for pine vs. 0.58 for spruce; z = 7.27, p < 0.001). Pine stands also had more than double as large bilberry plants as spruce stands (predicted mean dry mass: 0.36 vs 0.16 g; χ^2^ = 14.7, p < 0.001; Fig 3b). There was also a major difference in berry production in pine vs spruce stands at plot level (490 vs 5 berries in total; berries in 38 vs 1 plot, mean 7.45 vs 0.26, Fig 3c).

**Table 3:** Results summary for the mixed effect model analysis of the three bilberry performance variables.

	Dry mass (log_2_)			Cover		
	Estimate	z	p	Estimate	z	p
*Conditional model*						
Intercept (Pine)	−1.50	−5.82	<0.001	−0.02	−0.13	0.900
Spruce	−1.24	−2.72	0.007	−1.98	−7.30	<0.001
*Zero-inflation model*						
Intercept (Pine)				−2.75	−4.62	<0.001
Spruce				3.07	4.65	<0.001

Dry mass (size) was log_2_ transformed and analysed with Linear mixed effects model, and the table shows a difference in the mean dry mass between stand types. Cover (%) were analysed with zero-inflated generalised mixed models which tested for the probability of zero values between stand types (zero inflated model part), and for the mean difference between non-zero values (conditional part).

## Discussion

Our results showed that the soil organic carbon (SOC) present in forest soils did not depend on tree species identity under similar environmental conditions in production stands of spruce or pine, and that the patterns of SOC density between stand types only diverged with soil depth. The impact of tree species identity on performance of bilberry, however, was dramatic and spruce stands had substantially lower cover, plant size and berry production. This study thus indicates a lesser impact of tree species identity on carbon storage than expected, but a much stronger effect on the functional important bilberry in production forest than previously reported.

### Stand type effects on SOC density

In contrast to expectations and other studies [21,46], the relative amount of organic carboned stored in soil (SOC density), showed little overall difference between stand types of either spruce or pine. In a field experiment in Sweden where site productivity (medium fertility) and stand age (60 year) were accounted for in a similar way to our study, pine stands showed higher levels of SOC than spruce stands [29], whereas inventory-based large-scale studies have shown the opposite pattern [21]. Blaško et al [29] studied a medium productivity system where pine forest produced more aboveground biomass than spruce, and they suggested that pine stands therefore had higher amounts and different quality of litter, which may have give higher input of organic matter to soils and resulted in higher carbon storage. However, other Swedish studies who also controlled for productivity in their study design found that spruce stands had higher SOC-stocks than both pine and birch stands [20,46]. The variation in results across studies may also indicate that systems may differ in their carbon content independent of tree species identity, or that there are changes within stand types across time and space. However, we showed contrasting patterns in SOC density with soil depth dependent on tree species identity. The pine stands had highest SOC density at 5 cm, whereas it peaked at 15 cm depth in spruce stands. This concurs with several other studies and may be explained by relatively more litterfall from the more abundant understorey in pine forests [13,29; current study] or perhaps faster decomposition rates in spruce forests as moisture may be less limiting in such dense forests [47,48]. However, studies from other sites in Sweden have found higher SOC-levels in the upper humus layer [20] and perhaps pinpointing the need for further studies and an investigations into how different methods and calculations may bias results. In any case, the overall decline in SOC density that we found with depth in forest soils, independent of tree species identity, is in accordance with the semi-controlled studies from Swedish boreal forests [20,29], and most likely reflects the fact that upper soil layers in general have higher levels of humus due to the input of litter yet to be decomposed.

The variation in SOC-density across previous studies, as well as the current study, may also root in unexplained factors, or limitations to the approaches and methods used. When it comes to the latter there are many ways to estimate SOC. We used loss-on-ignition as basis for calculating SOM-% and converting this to a SOC-density, which is an easily accessible method that is cheap and reliable, and since all methods for C-calculations has its limitations, it is often recommended [36,38]. The use of conversion factors may also be criticized, but many studies point to 0.5 as a robust conversion from SOM to SOC-content [36] and combined with the use of area or volume based unit taking bulk density and soil type into account [49] we believe our C-density estimations are fairly robust. It is important to remember that the current study did not aim at calculating C-stocks at stand level but used the samples to compare stands of coniferous forest with identical soils, sediments and rock type, to test whether there was a relative difference in C-stock levels for pine and spruce stands. However, the results may be used, in combination with more data, to calculate such stand or landscape level C-stocks if required. Potentially, our standardised sampling at various depths (5, 15 and 25 cm) could include some bias related to the exact bordering of the horizons in the podzol soils, and many studies therefore take soil profiles into account. On the other hand, the borders were often not very distinct in our soil profiles, and we therefore decided to standardise sampling depth instead of adjusting these to soil profiles to avoid introducing the potential impact of subjective choices in our sampling.

### Bilberry performance

As predicted, the performance of bilberry was greatly enhanced in production stands of pine vs. those of spruce. However, the impact of tree species identity on bilberry performance was much stronger than we expected as indicated by the magnitude of difference in predicted cover values (10 x), plant size (>2 x), and berry production (100 x) between pine and spruce production stands. The difference in bilberry cover, for example, in production forest of pine (ca 50%) vs. spruce (ca. 5%) were much larger than reported in any other studies. Most other studies that compared bilberry performance with stand types did not control for confounding environmental factors through study design, but Hedwall et al. [12] that used Swedish forest inventory data found that abundance of all dwarf shrubs combined had decreased from a mean cover of 27–14% in hemiboreal and 38–32% in the boreal region from the 1950 until present. A regional study in southern Sweden found that bilberry cover peaked at ca. 40% in pine stands of 55-years, whereas bilberry cover in equivalent spruce stands was only ca. 5% but reached ca. 15% when stands were 80 years old [13; numbers interpreted from figures]. In old growth spruce forest in south-Norway dwarf shrub abundance have declined for several decades whereas large bryophyte cover have increased [50]. The high effect-size found in our study may reflect our semi-controlled study design that only targeted production stands with similar environmental conditions. Bilberry cover and ramet size appear to peak at some intermediate levels of light conditions in coniferous boreal forest [51,52]. Increased stand density and younger forest age may to a large extent explain the reduced light availability in many modern production forests [10,11,53]. In accordance with this, Hedwall and Brunet [54] found that light-demanding plant species had decreased most in abundance in a Swedish forest over a 20-year period and Eldegard et al. [51] found that bilberry cover peaked at almost double stand densities in Norwegian pine forest compared to spruce forest.

Bilberry reproduction was more was dramatically affected by stand type than expected. Spruce stands literally reduced berry production in our study plots to zero, whereas berry numbers in most pine stands were substantial. Impacts of land-use changes on plant reproduction is important to the long-term performance of plant populations, as well as for many interacting species in forest systems, but more rarely investigated in inventory-based studies. Several studies have pointed to light and temperature as decisive factors for flowering, pollination and berry production [55–57]. Eckerter et al. [55], for example, found that a range of reproductive variables in bilberry, such as flowering, seed set and berry production, was highest in forest gaps with high light availability. Although we only recorded berries in our study, it is likely that many of the light-induced changes in flower bud formation, bee-pollination, and seed and fruit set found by Eckerter, Buse (55) are also at play in the production stands studied here. In Finland it appears that bilberry yields, in the same way as for cover values, were generally higher in pine compared to spruce forest, and that increased forest density (i.e., basal area) explains reduced berry yields but mainly in spruce forest [58]. In our study, light intensity recorded by a lux meter was overall a fourfold higher in pine than spruce stands, whereas Petersson, Holmström (13) that utilised more precise measurements by help of spherical photos of the canopy, found that light transmittance to the forest floor was approximately doubled in pine stands. In any case, light appear to be reduced in spruce forests irrespectively of stem density and therefore it is likely that impact on light conditions comes from species differences in canopy and branching characteristics. Scots pine is characterised as having a relatively open canopy and is a light-demanding species, whereas Norway spruce have a thicker crown with branching further down towards the forest floor and considered a shade-tolerant species [59]. In sum, light conditions as a product of stand type (i.e., tree species identity) appeared to be the most likely mechanism that explained the negative impact of spruce production stands on bilberry performance in terms of size, cover and reproduction, and the magnitude of effects reported here appear unprecedented.

### Implications for nature management

Based on the assumption that SOC density reflected the relative differences in organic soil carbon in the studied production forest of spruce or pine, we may suggest that the tree species identity had minor impact on the forest carbon stocks when environmental conditions were similar as in our study. The discrepancy across different studies on which type of production forest system, Norway spruce or Scots pine dominated, that store carbon best [21,29] may call for a meta-study on the subject that better analyse the effect of tree species identity, spatio-temporal variables, and the interaction with other relevant management-related variables. Until then the neutral responses of SOC density to tree species identity in the current study may act as a guideline to management.

Spruce production stands, however, had a strong impact on the biodiversity component compared to pine stands, both the vegetative and reproductive components. Studies based on nation-wide forest inventory data in Fennoscandia have already showed large negative spruce-conversion effects on bilberry cover [12]. Our study may add that the land-use change of pine-to-spruce conversion on a stand scale may have impacted this functional important species even more dramatically than earlier reported. Not the least, our study included responses on plant size and reproduction often not investigated in large-scale inventory-based studies, and berry production was indeed the most radically impacted variable in spruce stands.

The mechanism behind these major differences in bilberry performance among stand types was most likely the strongly reduced light availability in spruce vs. pine production forest that in our controlled study was influenced largely by the denser canopy and branching of spruce, also highlighted by others [11]. From a management perspective, to reduce the negative effect on ecological function in production stands, forestry in these areas should manage forest for improved light availability by choosing Scots pine as regenerating species or manage Norway spruce stands with thinning at early regeneration stages to mimic light conditions in pine stands or old growth forest of spruce. Based on our findings Norwegian forestry policy should no longer subsidise denser and darker forest with spruce as dominating tree species either as a climate mitigation measure or to achieve a sustainable and ecologically sound forestry.

## Supporting information

S1 Appendix Stand type (Scots pine vs Norway spruce) effects on soil bulk density and soil organic matter. (XLSX)

S2 Appendix Data set used for the study. (DOCX)
