# Supplementary material for: Spruce vs. pine did not impact soil organic carbon density but strongly affected a functionally important dwarf shrub in a boreal production forest system
Source: PLoS One. 2025 Apr 15;20(4):e0320877. doi: 10.1371/journal.pone.0320877 (PMC11999120; doi:10.1371/journal.pone.0320877)
Supplement: S2 Appendix — (DOCX) [file pone.0320877.s002.docx]

# Appendix 1. Stand type (Scots pine vs Norway spruce) effects on soil bulk density and soil organic matter at various soil depths (5, 15, 25 cm) in ten paired stands in the Kaupanger forest, Western Norway


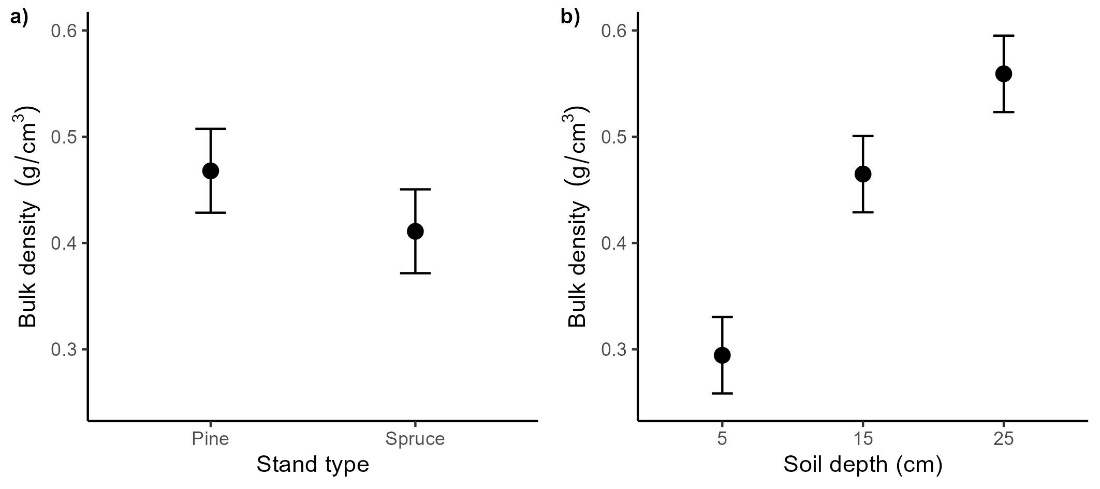


Fig S1: Bulk density (marginal mean) at a) the two stand types and b) the three soil depths, predicted from Linear mixed effects model with the non-significant interaction between the predictors removed. Error bars are 95% confidence intervals for means.


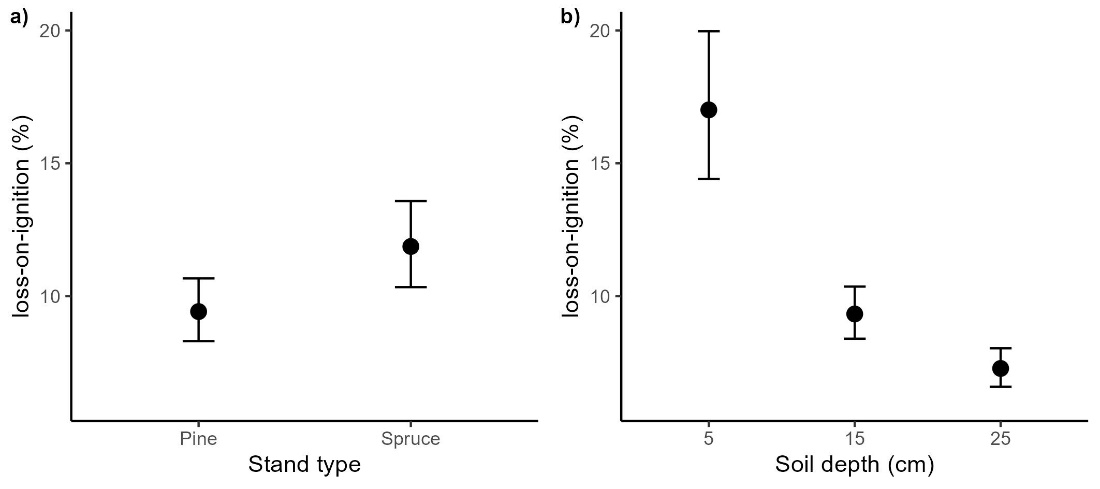


Fig S2: Soil organic matter (marginal mean) determined by loss-on-ignition at a) the two stand types and b) the three soil depths, predicted from a Generalised linear mixed effects model with the non-significant interaction between the predictors removed. Error bars are 95% confidence intervals for means.
